# Incarceration is a major risk factor for blood-borne infection among intravenous drug users: Incarceration and blood borne infection among intravenous drug users

**Published:** 2011-01-01

**Authors:** Mohammad Mehdi Mir-Nasseri, Ashraf MohammadKhani, Hamid Tavakkoli, Esmaeil Ansari, Hossein Poustchi

**Affiliations:** 1 Digestive Disease Research Center, Shariati Hospital, Tehran University of Medical Sciences, Tehran, IR Iran; 2 Al-Zahra University Hospital, Department of Gastroenterology, Isfahan University of Medical Sciences, Isfahan, IR Iran

**Keywords:** Hepatitis B Hepatitis C, HIV, Intravenous drug abuse, Prison

## Abstract

**Background:**

There is a strong association between hepatitis B virus (HBV), hepatitis C virus (HCV) and human immunodeficiency virus (HIV) infection which are mainly transmitted by contamination with blood via intravenous drug abuse (IVDU) or sexual contact.

**Objectives:**

To determine the prevalence of these infections and the risk factors associated with them among prisoner and non-prisoner IVDUs in Tehran, Iran.

**Patients and Methods:**

This cross-sectional study was performed in two jails and three drug rehabilitation centers between 2001 and 2002 in Tehran. HBsAg and HBcAb were checked using highly specific third generation enzyme immunoassays (DIA.PRO, Italy, specificity >99%, and Radim, Italy, specificity 99.7%, respectively). HCVAb was detected using ELISA (DIA.PRO, Italy) with both sensitivity and specificity >98%. HIVAb test (DRG Diagnostics kit, Germany) was performed for 459 of the 468 IDU subjects.

**Results:**

392 prisoners and 135 individual attending drug rehabilitation centers were approached. Of the 518 subjects studied, 464 (89.5%) were male, 386 (74.5%) were prisoners and 132 (25.5%) were non-prisoners. In this study, HBsAg, HCVAb and HIVAb were positive in 19 (3.7%), 359 (69.5%) and 70 (15.5%) of subjects, respectively. These tests were positive in 17 (4.5%), 311 (80.5%) and 63 (17%) among prisoners and 2 (1.5%), 48 (36.5%) and 7 (7.8%) in non-prisoners, respectively. Multiple logistic regression analysis revealed that independent factors related to co-infection of HCV and HIV infection were imprisonment (p<0.001. OR: 7.5) and using common syringe (p=0.03, OR: 4.5).

**Conclusions:**

Our findings strongly suggest that drug injection inside prison carries is a risk for HIV infection and that HIV infection among IDUs is likely to be bridged to the broader population through sexual contact without using effective prevention programs.

## Background

There is a strong association between hepatitis B virus (HBV), hepatitis C virus (HCV) and human immunodeficiency virus (HIV) infection [1][2]. All three infections are transmitted through intravenous drug abuse or sexual contact, although the latter route is not a usual way of HCV transmission [3][4][5]. Hepatitis A, B and C are considered as "desmoteric" infections, which mean their prevalence is generally higher in prisoners than in non-prisoners. Many studies showed that imprisonment is an important predictor of HBV, HCV and HIV infection [6][7][8][9][10]. Prison conditions increase the risk of transmission of infections, including blood-borne viral infections; the risk is further increased by the use of unsterile equipment used for injection [11]. Intravenous drug users (IDUs) are at a potential risk for acquiring blood-borne infections by parenteral and sexual routes. In many research, HBV, HCV and HIV infections have been found to coexist in IDUs [12][13][14][15][16]. However, there is scant information on the risk factors associated with these co-infections and their prevalence in Iran.

## Objectives

Given a large proportion of prisoners are drug addicts, we conducted this study to determine the prevalence of these infections and the risk factors associated with them among prisoner and non-prisoner IDUs in Tehran, Iran.

## Patients and Methods

This cross-sectional study was performed in two jails and three drug rehabilitation centers. The authorities of these places in Tehran were informed about the possible high rates of HBV and HCV infections among IVDUs and the potential risks and complications of HIV infection. In this study, an IVDU was defined as "a person using any kind of injectable drug (i.e., heroin, cocaine, opium, etc) regularly for at least one year." Prison inmates were selected at random; IVDUs attending the three reference drug treatment centers in Tehran were enrolled consecutively. Researchers met groups of 20-40 prisoners each time when the purpose and the nature of the study was fully explained for them. They were informed of the consequences of HBV, HCV and HIV infections. They were reassured that the study information would remain confidential and that participation in the study was voluntary. Prisoners were also approached individually, if so desired. After obtaining written informed consent from each subject, a questionnaire was completed to assess the probable risk factors for HBV, HCV and HIV infections. The questionnaire covered questions related to demography, socioeconomic status, marital status, level of education, occupation, pattern and type of intravenous drug used, duration of drug injection, use of shared injecting equipment, sexual behavior, traditional blood letting (hejamat), dental procedures, surgical operation, history of blood transfusion, ear piercing, and tattooing. After completing the questionnaire, 5 mL of blood was drawn from each subject and transferred into a sterile disposable Falcon tube. A similar identifier code was assigned to the questionnaire and blood sample. The blood samples were transported to the laboratory of the Digestive Disease Research Center in Shariati Hospital, Tehran within 2-5 hours at 2-8 °C. They were then centrifuged and sera were separated and transferred to a sterile tube with the same code. The sera were then stored at -70 °C until processing, which was done once the specimen collection was complete. HBsAg and HBcAb were checked using highly specific third generation enzyme immunoassays (DIA.PRO, Italy, specificity >99%, and Radim, Italy, specificity 99.7%, respectively). HCVAb was detected using ELISA (DIA.PRO, Italy) with both sensitivity and specificity >98%. HIV testing (DRG Diagnostics kit, Germany) was performed for 459 of the 468 IDU subjects. One HIV-positive subject was excluded from the study due to incomplete data. The tests were performed by one laboratory technician.

### Statistical analysis

SPSS® ver 15 for Windows® (SPSS Inc, Chicago, IL, USA) was used for all analyses. X2 and Fisher's exact tests were used to find the correlation between imprisonment and probable risk factors. Student's t-test and one-way ANOVA were used for comparison of means of continuous variables. Independent predictors of disease transmission were identified by multiple logistic regression analysis, with backward stepwise variable selection. A p<0.05 was considered statistically significant.

## Results

In this study 392 prisoners and 135 individual attending drug rehabilitation centers were approached. Of them, five prisoners and three non-prisoners refused to participate. Personal preference was the only reason to refuse participation in this study. One of the subjects was also check for HIV only and was therefore excluded from the study. Of the 518 subjects studied, 464 (89.5%) were men, 386 (74.5%) were prisoners and 132 (25.5%) were non-prisoners. HBsAg, HBcAb and HCVAb were checked for all 518 subjects; HIVAb was also tested in a subset of 458 subjects. Sixty subjects did not agree to test for HIV and were thus excluded for this test. In this study HBsAg, HCVAb and HIVAb were positive in 19 (3.7%), 359 (69.5%) and 70 (15.5%) of subjects, respectively. These tests was positive in 17 (4.5%), 311 (80.5%) and 63 (17%) among prisoners and 2 (1.5%), 48 (36.5%) and 7 (7.8%) in non-prisoner, respectively. The prevalence of Confection with HBsAg+ and HCVAb+, HBsAg+ and HIVAb+, HCVAb+ and HIVAb+ and triple infection with HBV, HCV and HIV is presented in Table 1. The prevalence of co-infection was significantly higher in prisoners in all the studied groups. In the next step, we sought to compare risk factors for disease transmission in these two groups. As presented in Table 2, all known risk factors related to infection transmission are significantly higher in prisoners compared to non-prisoners (p<0.001 in all variables). Finally, multiple logistic regression analysis revealed that independent factors related to co-infection of HCV and HIV were "imprisonment" (p<0.001; OR: 7.5) and using common syringe (p = 0.03; OR: 4.5).

**Table 1:** Co-infection of viral markers in prisoners and non-prisoners

**Viral markers**	**Total **(%)	**Prisoner **(%)	**Non-prisoner **(%)
**HBSAg (+), HCVAb (+)**	16 (3.1)	16 (4.1)	0
**HBSAg (+), HIVAb (+)**	3 (0.6)	3 (0.8)	0
**HCVAb (+), HIVAb (+)**	58 (11.2)	56 (14.5)	2 (1.5)
**HBSAg (+), HCVAb (+), HIVAb (+)**	3 (0.6)	3 (0.8)	0

**Table 2:** Comparison of risk factors for disease transmission between prisoners and non-prisoners

**Variables**	**Totaln **(%)	**Prisonern **(%)	**Non-prisonern **(%)	**p-value**
**Mean age **(year)	35.24	35.85	33.45	0.007 a
**Gender**				
**M**	464 (89.6)	336 (72)	128 (28)	0.001 a
**F**	54 (10.4)	50 (93)	4 (7)	
**Mean duration of IVDU **(year)	4.533	4.78	3.78	0.02 b
**History of sharing needle**	321 (62)	255 (79 )	66 (21)	0.001 b
**History of tattooing**	272 (52.5)	230 (85)	42 (15)	<0.001 b
**History of heterosexual**	272 (52.5)	182 (67)	90 (23)	0.001 b
**History of homosexual**	53 (10.21)	27 (51)	26 (49)	<0.001 b
**Bisexual**	49 (9.5)	23 (47)	26 (53)	<0.001 b

^a^ Independent samples Student's t test

^b^ χ(2) test

## Discussion

To better understand the risk of blood-borne infection in relation to imprisonment in IVDUs, we evaluated the prevalence of HBV, HCV and HIV and also co-infection among IVDUs in prisons and IVDUs who attended drug rehabilitation centers and compared risk factors of disease transmission between these two groups. We found that blood-borne infection and co-infection of the studied diseases are significantly more prevalent among IVDUs than general population of Iran which is mainly due to risky behaviors in the latter group. In this study, we were able to show that independent predictors of HCV and HIV co-infection are history of sharing needle and imprisonment and that each of which increases the risk of co-infection. This result indicates that imprisonment increases the risk of co-infection by 7.5-fold. This can be attributed to the higher risky behaviors during the incarceration including using common needle, razors, and even practicing unsafe sexual behaviors. This hypothesis is confirmed in several other studies. In a study by Zamani, et al, [17] on IVDU participants, infection was strongly associated with a history of shared drug injection inside prison, whereas it was not significantly associated with shared drug injection outside prison. Similar findings have been reported from Thailand, where drug injecting inside prison was shown to associate with HIV infection among prisoners [18]. The risk of disease transmission is also related to the length and number of incarceration as confirmed in a study that length of incarcerations increased the risk of HCV infection by 3.5-fold [19]. In another study among community based drug users in Tehran, the prevalence of HIV infection was 23.2% among male IVDUs. In a multivariate analysis, a history of shared drug injection inside prison (OR: 2.5) and multiple incarcerations (OR: 3.13) were associated with a significantly higher prevalence of HIV infection [20]. Clinically, co-infection of HBV with HIV and HCV with HIV is also important. The natural history of chronic hepatitis B and C is altered by simultaneous infection with HIV. Immune control of HBV is negatively affected by HIV leading to reduction of HBsAg clearance. Furthermore, HIV increases the HBV viral load [21]. In untreated HIV population, faster progression to liver cirrhosis is reported for HBV/HIV patients [22]. Furthermore, hepatocellular carcinoma may develop at a younger age and with a more aggressive presentation [23]. Co-infection of HCV with HIV is also a major public health problem. Of the 33.4 million HIV-infected individuals in the world in 2008, it is estimated that at least five million are co-infected with HCV. Recent data from the United State of America indicate that, 25%-30% of patients with HIV are co-infected with HCV [24] reflecting the contribution of at high risk population such as prison inmates. Also, 65%-70% of HIV-positive prisoners in the US are co-infected with HCV compared to 18%-25% of HIV-positive in general population [25]. In the natural course of HCV, it is evident that co-infection with HIV reduces the immune response to HCV and diminishes the chance of spontaneous clearance. Many studies revealed that the presence of HIV in HCV subjects leads to faster disease progression and liver failure [26]. It is therefore clear that co-infection of these three blood-borne infections is a serious epidemiological and clinical problem worldwide as well as Iran. These diseases can easily transferred among high risk population, especially in prisons where many individual with different diseases are kept in places with low hygiene. Therefore, harm reduction programs should urgently be expanded in prisons and in correctional centers to prevent transmission of these diseases to susceptible persons.Prevalence of infection with hepatitis B and C is high in those with high risk behaviors including IVDUs and persons with unsafe sex practice particularly among prisoners. This can be more complicated and increase the burden of diseases by co-infection with HIV. Therefore, it is imperative to implement some strategies like harm reduction program to reduce the burden of blood-borne infection among prisoners which directly influences the transmission of these diseases to the community.

## References

[1] Levine OS, Vlahov D, Koehler J, Cohn S, Spronk AM, Nelson KE, Levine OS (1995). Seroepidemiology of hepatitis B virus in a population of injecting drug users. Association with drug injection patterns. Am J Epidemiol.

[2] Tor J, Llibre JM, Carbonell M, Muga R, Ribera A, Soriano V, Clotet B, Sabria M, Foz M (1990). Sexual transmission of hepatitis C virus and its relation with hepatitis B virus and HIV. BMJ.

[3] Muller R, Stark K, Guggenmoos-Holzmann I, Wirth D, Bienzle U (1995). Imprisonment: a risk factor for HIV infection counteracting education and prevention programmes for intravenous drug users. AIDS.

[4] Hull HF, Lyons LH, Mann JM, Hadler SC, Steece R, Skeels MR (1985). Incidence of hepatitis B in the penitentiary of New Mexico. Am J Public Health.

[5] Tibbs CJ (1995). Methods of transmission of hepatitis C. J Viral Hepat.

[6] Pallas J, Farinas-Alvarez C, Prieto D, Llorca J, Delgado-Rodriguez M (1999). Risk factors for monoinfections and coinfections with HIV, hepatitis B and hepatitis C viruses in northern Spanish prisoners. Epidemiol Infect.

[7] Alizadeh AH, Alavian SM, Jafari K, Yazdi N (2005). Prevalence of hepatitis C virus infection and its related risk factors in drug abuser prisoners in Hamedan, Iran. World J Gastroenterol.

[8] Davies AG, Dominy NJ, Peters A, Bath GE, Burns SM, Richardson AM (1995). HIV in injecting drug users in Edinburgh: prevalence and correlates. J Acquir Immune Defic Syndr Hum Retrovirol.

[9] Vlahov D, Nelson KE, Quinn TC, Kendig N (1993). Prevalence and incidence of hepatitis C virus infection among male prison inmates in Maryland. Eur J Epidemiol.

[10] Gaughwin MD, Douglas RM, Liew C, Davies L, Mylvaganam A, Treffke H, Edwards J, Ali R (1991). HIV prevalence and risk behaviours for HIV transmission in South Australian prisons. AIDS.

[11] Mirnaseri SMM, Poustchi H, Naseri Moghadam S, Nouraei S.M, Tahaghoghi S, Afshar P, Mohammad Khani A, Tavakoli H, Malekzadeh R (2005). HCV in intravenous drug users. Govaresh J.

[12] Patti AM, Santi AL, Pompa MG, Giustini C, Vescia N, Mastroeni I, Fara GM (1993). Viral hepatitis and drugs: a continuing problem. Int J Epidemiol.

[13] Vahdani P, Hosseini-Moghaddam SM, Family A, Moheb-Dezfouli R (2009). Prevalence of HBV, HCV, HIV and syphilis among homeless subjects older than fifteen years in Tehran. Arch Iran Med.

[14] Alavian SM, Gholami B, Masarrat S (2002). Hepatitis C risk factors in Iranian volunteer blood donors: a case-control study. J Gastroenterol Hepatol.

[15] Allwright S, Bradley F, Long J, Barry J, Thornton L, Parry JV (2000). Prevalence of antibodies to hepatitis B, hepatitis C, and HIV and risk factors in Irish prisoners: results of a national cross sectional survey. BMJ.

[16] Rowhani-Rahbar A, Tabatabaee-Yazdi A, Panahi M (2004). Prevalence of common blood-borne infections among imprisoned injection drug users in Mashhad, North-East of Iran. Arch Iran Med.

[17] Zamani S, Kihara M, Gouya MM, Vazirian M, Ono-Kihara M, Razzaghi EM, Ichikawa S (2005). Prevalence of and factors associated with HIV-1 infection among drug users visiting treatment centers in Tehran, Iran. AIDS.

[18] Vanichseni S, Kitayaporn D, Mastro TD, Mock PA, Raktham S, Des Jarlais DC, Sujarita S, Srisuwanvilai LO, Young NL, Wasi C, Subbarao S, Heyward WL, Esparza L, Choopanya K (2001). Continued high HIV-1 incidence in a vaccine trial preparatory cohort of injection drug users in Bangkok, Thailand. AIDS.

[19] Zamani S, Ichikawa S, Nassirimanesh B, Vazirian M, Ichikawa K, Gouya MM, Afshar P, Ono-Kihara M, Ravari SM, Kihara M (2007). Prevalence and correlates of hepatitis C virus infection among injecting drug users in Tehran. Int J Drug Policy.

[20] Zamani S, Kihara M, Gouya MM, Vazirian M, Nassirimanesh B, Ono-Kihara M, Ravari SM, Safaie A, Ichikawa S (2006). High prevalence of H incarceratioIV infection associated withn among community-based injecting drug users in Tehran, Iran. J Acquir Immune Defic Syndr.

[21] Hadler SC, Judson FN, O'Malley PM, Altman NL, Penley K, Buchbinder S, Schable CA, Coleman PJ, Ostrow DN, Francis DP (1991). Outcome of hepatitis B virus infection in homosexual men and its relation to prior human immunodeficiency virus infection. J Infect Dis.

[22] Puoti M, Zanini B, Quinzan GP, Ravasio L, Paraninfo G, Santantonio T, Rollo A, Artioli S, Maggiolo F, Zaltron S, Raise E, Mignani E, Resta F, Verucchi G, Pastore G, Suter F, Carosi G, MASTER HIV/HCV Co-Infection Study Group (2004). A randomized, controlled trial of triple antiviral therapy as initial treatment of chronic hepatitis C in HIV-infected patients. J Hepatol.

[23] Puoti M, Bruno R, Soriano V, Donato F, Gaeta GB, Quinzan GP, Precone D, Gelatti U, Asensi V, Vaccher E, HIV HCC Cooperative Italian-Spanish Group (2004). Hepatocellular carcinoma in HIV-infected patients: epidemiological features, clinical presentation and outcome. AIDS.

[24] Singal AK, Anand BS (2009). Management of hepatitis C virus infection in HIV/HCV co-infected patients: clinical review. World J Gastroenterol.

[25] Weinbaum CM, Sabin KM, Santibanez SS (2005). Hepatitis B, hepatitis C, and HIV in correctional populations: a review of epidemiology and prevention. AIDS.

[26] Danta M, Semmo N, Fabris P, Brown D, Pybus OG, Sabin CA, Bhagani S, Emery VC, Dusheiko GM, Klenerman P (2008). Impact of HIV on host-virus interactions during early hepatitis C virus infection. J Infect Dis.

